# Long‐Term Impact of Orthodontic Treatment on Oral Behaviours, Temporomandibular Disorder‐Related Pain, and Anxiety: An 18‐Month Prospective Study

**DOI:** 10.1111/ocr.70030

**Published:** 2025-09-12

**Authors:** Bachar Reda, Giovanna Zanon, Luca Contardo, Mohammed Nahidh, Mariam Hmeidan

**Affiliations:** ^1^ Department of Medical, Surgical and Health Sciences, School of Dentistry University of Trieste Trieste Italy; ^2^ Department of Orthodontics, College of Dentistry University of Baghdad Baghdad Iraq; ^3^ Institut National de Santé Publique, d'Epidémiologie Clinique et de Toxicologie‐Liban (INSPECT‐LB) Beirut Lebanon

**Keywords:** generalised anxiety disorder‐7, oral behaviours checklist‐21, orthodontic treatment, orthodontics, temporomandibular disorder

## Abstract

**Objective:**

Orthodontic treatment is a common approach for correcting malocclusion but is often associated with discomfort. The aim of this study was to assess the longitudinal changes in oral behaviours, temporomandibular disorder (TMD)‐related pain and anxiety among university students undergoing orthodontic treatment compared to untreated controls.

**Materials and Methods:**

A prospective cohort study was conducted at the University of Trieste, Italy. Participants were grouped based on the presence or absence of active orthodontic treatment and asked to complete an electronic survey comprising the Oral behaviour checklist‐21 (OBC‐21), TMD pain screener and generalised anxiety disorder‐7 (GAD‐7) at baseline (T0), after 6 months (T1), 12 months (T2) and 18 months (T3). Repeated measures ANOVA analysed within‐group changes over time, while mixed ANOVA assessed group–time interactions.

**Results:**

A total of 114 participants completed all follow‐ups, with 57 in each group and comparable sex distribution. In the orthodontic group, no significant changes were observed across the three measures (*p* > 0.05). The non‐orthodontic group showed a slight increase in OBC‐21 scores after 12 months (*p* = 0.034) and a decrease in GAD‐7 scores after 18 months (*p* = 0.048). Mixed ANOVA confirmed the absence of significant changes in the pattern of scores between the orthodontic and non‐orthodontic groups over time (*p* > 0.05).

**Conclusion:**

Orthodontic treatment does not significantly influence oral behaviours, TMD‐related pain or anxiety over time. Routine screening for these factors in orthodontic patients is not required as a standard practice for all orthodontic patients; instead, individualised assessment should be based on clinical indications.

## 1 Introduction

Orthodontics is a field of dentistry focused on correcting malocclusions to achieve functional and aesthetic improvements [1]. Treatment approaches involve the use of fixed or removable appliances that correct dental alignment and occlusion either immediately or progressively over time [2]. However, these appliances may introduce adverse effects including oral discomfort, altered masticatory function, compromised oral hygiene and psychological burdens during daily interactions [3, 4, 5, 6].

The severity and nature of these side effects vary depending on the type of orthodontic appliance used [7, 8]. For instance, lingual orthodontics offers aesthetic advantages but tends to cause more discomfort and technical challenges, including speech difficulty [9, 10, 11]. In contrast, aligner treatment is associated with lower reported discomfort levels compared to fixed appliances, with the latter group exhibiting higher analgesic consumption [12]. For patients undergoing fixed orthodontic therapy, the first 4 weeks after treatment initiation typically result in greater discomfort [13], although individual responses may vary [14].

While physical discomfort during orthodontic treatment is well‐documented, its potential impact on behavioural and psychological factors remains inconclusive. Oral behaviours, including clenching, grinding and nail biting, may both influence and be influenced by orthodontic therapy. However, existing evidence on this relationship is contradictory. One study reported the absence of correlation between oral behaviours, as evaluated by the Oral Behaviour Checklist‐21 (OBC‐21), and facial divergence in either adolescents or adults [15]. In contrast, another study has reported that children with Class II malocclusion were more likely to engage in behaviours such as clenching, grinding and pencil or nail biting [16]. When correcting malocclusion through orthodontic interventions, one study has identified that 25% of patients undergoing orthodontic treatment had high‐risk grades of oral behaviours [17].

As for the association between orthodontic treatment and the incidence of temporomandibular disorders (TMDs), evidence remains inconclusive. A systematic review has concluded that patients receiving orthodontic treatment were approximately two times more likely to develop TMD [18], whereas other studies stated the lack of association [19, 20]. Although one recent study reported improvement in temporomandibular joint (TMJ) health following orthodontic treatment [21], claims in the literature suggesting that orthodontic treatment may cause or cure TMD remain controversial and should be critically evaluated and supported by robust evidence [22].

Similarly, psychological factors such as anxiety have been found to correlate with experimentally induced orthodontic pain when compared with a control group [23]. A previous study indicated that anxiety is prevalent among patients undergoing orthodontic treatment, especially in the early stages of the therapy [24], with no significant differences observed between patients using fixed appliances and those using aligners [25]. While anxiety levels were reported to significantly decrease after the first month of treatment [26]. However, the absence of longitudinal follow‐up restricts the understanding of the long‐term progression of anxiety during orthodontic treatment.

Psychological frameworks, such as anxiety models, underscore the influence of emotional states on physiological responses and behavioural adaptations, which may contribute to the onset or exacerbation of oral behaviours and TMDs [27, 28]. Furthermore, biopsychosocial models of TMD diagnosis adopted by the Diagnostic Criteria for TMD (DC/TMD) emphasise the interplay of biological, psychological and social factors in symptom development and persistence [29, 30]. Given the conflicting evidence regarding these relationships during orthodontic treatment, longitudinal controlled studies are essential to clarify these complex interactions.

Therefore, the aim of this study was to explore longitudinal changes in oral behaviours, TMD‐related pain and generalised anxiety in university students receiving orthodontic treatment, compared to an untreated control group. The rationale behind selecting these variables is their potential relevance to orthodontic treatment, with insufficient data on their longitudinal interactions.

## 2 Materials and Methods

### 2.1 Study Design and Settings

This prospective longitudinal cohort study was conducted at the University Stomatological Clinic of Trieste's Maggiore Hospital between January 2021 and June 2022. The target population comprised university students undergoing fixed multibracket orthodontic treatment for aligning and levelling, with a comparable sample size not undergoing any orthodontic treatment. Ethical approval was obtained from the institutional review board of the Ateneo Università degli Studi di Trieste (IRB ID: n. 89/11062018). To be included, participants from both groups must provide their full consent to participate in this study through a dedicated internal site of the University of Xxxx, specifically designed for privacy‐related matters. Participants with current use of medications influencing behaviours, such as antidepressants, and those who did not comply with follow‐up assessments at the beginning of the study (T0), after 6 months (T1), 12 months (T2) and 18 months (T3) were excluded from the study. Data for both groups were collected concurrently to reduce the risk of temporal bias. Recruited participants were categorised into two groups: Group 1 (active orthodontic treatment) and Group 2 (control group; no orthodontic treatment), without specific information on treatment duration or phase.

### 2.2 Sample Size Calculation

To detect within‐group, between‐group and interaction effects across the four time points (T0, 6 months, 12 months and 18 months), a sample size calculation was performed using G*Power version 3.1 (Heinrich‐Heine‐University Düsseldorf, Düsseldorf, Germany). Based on a medium effect size (*f* = 0.25), an alpha level of 0.05, and a desired power of 0.80, the required sample size was estimated at 24 participants per group, totalling 48 participants. To enhance the power of the study and the generalisability of the findings, the sample size was further increased, and a total of 57 participants per group was included.

### 2.3 Study Instruments

The questionnaire was developed using the REDCap (Research Electronic Data Capture) platform, an internal system of the University of Trieste dedicated to the Department of Surgical and Medical Sciences. Participants were required to indicate whether they were undergoing orthodontic treatment, along with their sex. The remaining questionnaire included the OBC‐21, TMD pain screener and generalised anxiety disorder–7 (GAD‐7). Participants were required to fill out the questionnaire at the beginning of the study (T0), after 6 months (T1), 12 months (T2) and 18 months (T3).

OBC‐21 was developed to help clinicians identify oral behaviours as a part of Axis II DC/TMD [29], and has been validated in multiple languages including Italian [31]. This survey comprised 21 questions, two addressing nocturnal activities and 19 focusing on daytime behaviours. Each question offered five response options: ‘Never’ is scored as 0, ‘< 1 night/month’ as 1, ‘1–3 nights/month’ as 2, ‘1–3 nights/week’ as 3 and ‘4–7 nights/week’ as 4. The maximum achievable score was 84 [29, 32, 33].

TMD pain screener: Part of Axis I in the DC/TMD [29]. This reliable and valid self‐reported instrument is used to screen for pain‐related TMD and allows clinicians to detect patients with painful TMD in a fast and cost‐effective approach [34]. The TMD pain screener comprised six questions, the first question dedicated to the duration of pain with answers no pain (0 points), pain comes and goes (1 point) and pain is always present (2 points). The remaining five questions had yes/no answers, where yes granted the patients 1 point. A total score > 3 suggested the presence of TMD [32, 33]. In this study, the TMD pain screener total score was treated as a continuous variable to assess symptom fluctuations over time, with assumptions for parametric analysis verified prior to applying mixed‐model ANOVA.

GAD‐7: Used for assessing the presence and degree of anxiety. This reliable and validated self‐reported tool is widely used and is included as a part of the Axis II DC/TMD [29, 35, 36], and has been validated among the Italian population [37]. Participants were required to fill out seven questions reflecting the frequency of anxiety symptoms over the past 2 weeks with answers including ‘not at all’ (0), ‘several days’ (1), ‘more than half the days’ (2) and ‘nearly every day’ (3). The GAD‐7 total score ranged from 0 to 21, with 0–4 qualified as minimal anxiety, 5–9 as mild, 10–14 as moderate and 15–21 as severe [33, 35].

### 2.4 Statistical Analysis

Data were analysed using SPSS version 26 (IBM Corp., Armonk, NY, USA). Descriptive statistics were reported using means and standard deviations for continuous variables, and frequencies and percentages for categorical variables. Shapiro–Wilk was performed to assess normality of the continuous variables for each scale within each group alone and for the whole study population together. To assess differences in OBC‐21, TMD pain screener and GAD‐7 over time within each group alone, repeated measures ANOVA was used for normally distributed variables and the Friedman test for those not exhibiting normal distribution. To assess the within‐subject, between‐subject and interaction effects between orthodontic intervention and time, repeated measures mixed‐model ANOVA was used. A *p*‐value < 0.05 was considered statistically significant.

## 3 Results

**FIGURE 1:**
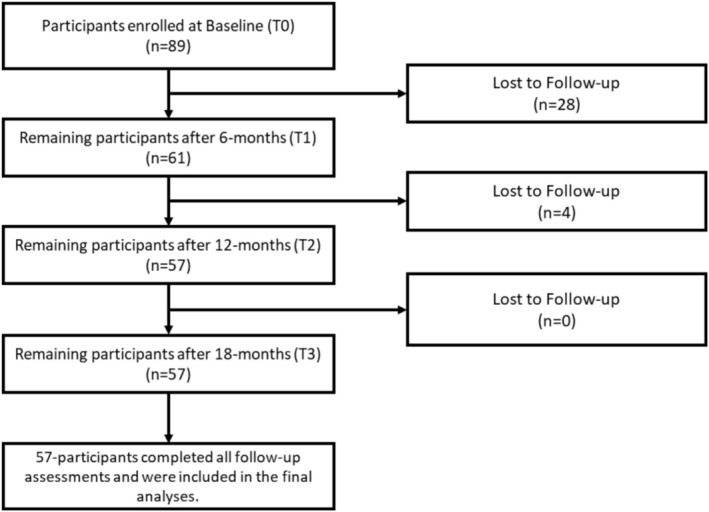
Participant flowchart for the orthodontic group.

At the beginning of the study, 89 participants undergoing active orthodontic treatment were enrolled. Over the follow‐up period, this number declined, resulting in 57 participants by the final assessment (Figure 1). To maintain group comparability, an equal number of participants (*n* = 57) were randomly selected from the control group using a computer‐generated random number sequence. In total, 114 participants completed the follow‐up assessments and were included in this study, with 57 individuals per group. In Group 1 (active orthodontic treatment), 72% were females and 28% were males. Comparably, in Group 2 (control group; no orthodontic treatment), 68.4% were females and 31.6% were males. The mean age of the entire sample was 24.1 (SD = 5.9).

OBC‐21: Mean OBC‐21 scores were comparable across both groups at T0, T1, T2 and T3, as detailed in Table 1. Data for both groups was normally distributed and repeated measures ANOVA was used for within‐group comparison (Table 2). In Group 1, no significant change in the OBC‐21 scores was found over time. However, in Group 2, a significant within‐group effect was observed (*p* = 0.03; Table 2). Pairwise comparison indicated a significant increase of 2.28 points from T0 (baseline) to T2 (after 12 months). Mixed ANOVA revealed a lack of within‐subject effect over time across the whole sample, no significant difference in average OBC‐21 scores between the two groups, and no interaction between time and group, indicating that changes in OBC‐21 scores over time were not significantly different between the groups (Table 2).

**TABLE 1:** Descriptive statistics for OBC‐21, TMD pain screener and GAD‐7 for orthodontic and non‐orthodontic groups.

Measures	Time	Orthodontic group mean (±SD)	Non‐orthodontic group mean (±SD)
OBC‐21 (Range 0–84)	T0	24.77 ± 10.40	21.67 ± 7.92
	T1	24.35 ± 8.33	22.82 ± 8.18
	T2	24.96 ± 7.64	23.95 ± 7.59
	T3	25.11 ± 8.38	22.46 ± 6.71
TMD pain screener (Range 0–6)	T0	1.42 ± 1.67	1.33 ± 1.70
	T1	1.40 ± 1.90	1.37 ± 1.78
	T2	1.40 ± 1.83	1.18 ± 1.72
	T3	1.44 ± 1.81	0.98 ± 1.48
GAD‐7 (Range 0–21)	T0	7.93 ± 4.52	8.95 ± 5.31
	T1	8.14 ± 5.02	7.88 ± 5.41
	T2	7.81 ± 5.20	7.42 ± 5.05
	T3	7.21 ± 4.58	7.11 ± 4.79

Abbreviations: GAD‐7, generalised anxiety disorder‐7; OBC‐21, oral behaviour checklist‐21; SD, standard deviation; T0, baseline; T1, 6 months; T2, 12 months; T3, 18 months; TMD pain screener, temporomandibular disorder pain screener.

**TABLE 2:** Normality tests, within group longitudinal analysis for orthodontic and non‐orthodontic groups, and mixed ANOVA results for the entire sample.

Measures	Group	Normality tests		Within group longitudinal analysis		Mixed ANOVA	
		Shapiro–Wilk *p*	Normality assumed?	Test used	*p*	Effect	*p*
OBC‐21	Orthodontic Group (1)	T0: 0.001 T1: 0.390 T2: 0.241 T3: 0.532	Yes	Repeated measures ANOVA	0.78	**Time** (Within) **Group** (Between) **Time** × **Group** (Interaction)	0.201 0.131 0.670
	Non‐orthodontic Group (2)	T0: 0.893 T1: 0.353 T2: 0.769 T3: 0.676	Yes	Repeated measures ANOVA	**0.03**		
TMD pain screener	Orthodontic Group (1)	T0: < 0.001 T1: < 0.001 T2: < 0.001 T3 < 0.001	No	Friedman	0.92	**Time** (Within) **Group** (Between) **Time** × **Group** (Interaction)	0.623 **0.005** 0.524
	Non‐Orthodontic Group (2)	T0: < 0.001 T1: < 0.001 T2: < 0.001 T3: < 0.001	No	Friedman	0.16		
GAD‐7	Orthodontic Group (1)	T0: 0.256 T1: 0.161 T2: 0.062 T3: 0.018	Yes	Repeated measures ANOVA	0.19	**Time** (Within) **Group** (Between) **Time** × **Group** (Interaction)	**0.005** 0.937 0.194
	Non‐orthodontic Group (2)	T0: 0.024 T1: 0.001 T2: 0.008 T3: 0.006	No	Friedman	**0.048**		

*Note:* Normality assessed using Shapiro–Wilk Test; *p* < 0.05 indicates deviation from normality. Bold *p*‐values indicate statistical significance at *p* < 0.05.

Abbreviations: GAD‐7, generalised anxiety disorder‐7; OBC‐21, oral behaviour checklist‐21; T0, baseline; T2, 12 months; T3, 18 months; TMD pain screener, temporomandibular disorder pain screener.

TMD pain screener: Mean TMD pain screener scores showed slight variations over time (Table 1). The distribution for both groups was not normal, and the Friedman test suggested the absence of significant within‐group changes over time (Table 2). Mixed ANOVA demonstrated no significant within‐subject effect for the whole sample over time, but there was a significant between‐group effect (*p* = 0.005), indicating average score differences between the groups. The interaction effect between time and the two groups was not significant (*p* = 0.52), suggesting that the pattern of change over time did not differ between the groups (Table 2).

GAD‐7: Descriptive statistics (means ± SD) for GAD‐7 scores at all time points are presented in Table 1. For Group 1, GAD‐7 scores were normally distributed, but repeated measures ANOVA showed no significant change over time (Table 2). In contrast, data were not normally distributed for Group 2, and the Friedman test highlighted a significant overall change in GAD‐7 scores over time (*p* = 0.048; Table 2). Pairwise comparison indicated a significant decrease of 1.84 points from T0 (baseline) to T3 (after 18 months). Mixed measures ANOVA showed a significant overall change in the GAD‐7 scores over time for the entire sample (*p* = 0.005), but there were no significant between‐group differences or interaction effects (Table 2).

## 4 Discussion

The aim of this longitudinal cohort study was to assess the impact of orthodontic treatment on oral behaviours, TMD‐related pain and generalised anxiety among university students. The rationale behind this study was to provide a comprehensive insight into the effect of orthodontic treatment on those aspects compared to a non‐orthodontic counterpart.

Oral behaviours were evaluated using the validated OBC‐21 scale. No significant changes over time were observed within the orthodontic group, whereas changes in the non‐orthodontic group were recorded, indicating the absence of a relationship between orthodontic treatment and the likelihood to engage in oral behaviours such as clenching, grinding and chewing food on one side only. The increase in OBC scores observed in the non‐orthodontic group is likely due to normal variability or transient factors such as stress, rather than clinically meaningful behavioural changes. Mixed ANOVA confirmed the absence of a significant interaction between orthodontic treatment and changes in OBC‐21 scores over time when compared to the non‐orthodontic group. While no studies have assessed the longitudinal impact of orthodontic treatment on oral behaviours as per the OBC‐21, a recent study reported a high prevalence of bruxism among orthodontic patients after 6 months of treatment [38], though it did not follow patients over time or include a control group to confirm the association. Conversely, another study reported a negligible impact of aligners on the frequency of awake bruxism [39]. Additionally, an observational study highlighted the need to screen for oral parafunctions for patients undergoing orthodontic treatment, noting that 25% have high‐risk grades of oral behaviours [17]; however, our findings showed similar mean OBC‐21 scores between both orthodontic and non‐orthodontic groups. This aligns with findings from a study on general Italian dental patients, which reported that 13.7% were also classified as high risk [40], reinforcing that orthodontic treatment is not a determining factor for increased engagement in oral behaviours.

As for TMD‐related pain, no significant changes over time were observed within either the orthodontic or the non‐orthodontic groups. However, a significant change over time was noted when assessing both groups together, suggesting that the observed variation is independent of orthodontic treatment. The absence of interaction between the two groups over time further confirmed the lack of significant change in the pattern of scores between the two groups. Our study results are in parallel to a previous case–control study that reported no association between TMD and orthodontic treatment [19]. Moreover, a systematic review concluded that orthodontic treatment had a negligible effect on the incidence of TMD [41], supporting our findings while emphasising the importance of screening for TMD symptoms and potential complications among orthodontic patients [17, 41].

No significant change in GAD‐7 scores was recorded within the orthodontic group, whereas the non‐orthodontic control group showed a reduction in total anxiety levels after 18‐month follow‐up. Our study findings suggest the lack of a significant association between orthodontic treatment and anxiety, while another study reported increased anxiety during the early stages of orthodontic treatment [24]. The discrepancy with our results may be explained by the timing of the assessment, as none of our study participants were at the initiation phase of treatment at baseline assessment, but rather they were already undergoing active orthodontic therapy. In parallel to our findings, a pilot study stated the lack of impact of the orthodontic system on anxiety levels [42]; instead, orthodontic treatment can enhance mental health status and improve the patients' attitude towards body image [43].

This study is the first to investigate the long‐term effects of orthodontic interventions on key dental and psychological components, including oral behaviours, TMD‐related pain and anxiety. The longitudinal cohort design strengthens the validity of the findings. Furthermore, the sample size exceeded the calculated minimum sample required, thereby improving the statistical power and enhancing the generalisability of the results. A balanced distribution of male and female participants between the two groups helped control for potential sex‐related confounding effects. The use of validated assessment scales contributed to the reliability and internal validity of the measurements. An additional methodological strength in this study was the decision to analyse these scales in their original continuous form, which minimised the risk of misclassification bias.

From a clinical perspective, this study supports the safety of orthodontic interventions in relation to oral behaviours, TMD‐related pain and anxiety. The findings highlight the importance of individualised assessment over routine screening for these factors, emphasising the need to focus on scientifically proven fundamental factors when evaluating orthodontic patients such as biomechanics, periodontal health, compliance and duration of treatment. Those findings can provide valuable insights for dentists across varying specialties. Although orthodontists appear to be aware of the lack of association between TMD and orthodontic treatment, other dental specialties have reported lower levels of knowledge in this concern [20], suggesting the need to improve awareness towards evidence‐based literature.

Although the survey instruments used in this study are reliable and validated, their subjective self‐reporting nature introduces the risk of response bias. Additionally, data collection through online surveys may have contributed to selection bias. The study's focus on university students limits the generalisability of the findings to broader populations. While the sample size was adequate and sex distribution was balanced between the two groups, other factors such as type and stage of orthodontic treatment, demographics and socioeconomic status, environmental factors or personality traits such as stress were not accounted for, resulting in the risk of potential confounding. Additionally, a 36% attrition rate in the orthodontic group and the use of complete‐case analysis without imputation may introduce bias if data were not missing at random, though balanced retention in the control group mitigates differential attrition effects. As the TMD pain screener is primarily a diagnostic tool not designed to measure symptom intensity, interpreting its total score as a continuous variable may have limitations; however, this approach preserves more information than a binary classification and enables detection of subtle changes or differences between groups. Future studies should consider more detailed symptom assessment methods and examine functional and non‐functional oral behaviours separately.

## 5 Conclusion

The findings of this study do not indicate a significant association between orthodontic treatment and oral behaviours' engagement, TMD‐related pain or the development or exacerbation of anxiety. These results highlight the importance of raising awareness about the lack of a significant relationship between orthodontic treatment and these factors, thereby supporting the use of individualised assessments rather than routine screening for orthodontic patients.

## Author Contributions

Concept and design aspects were led by B.R. and M.H. Data acquisition and statistical analysis were conducted by B.R. and M.H. Analysis and interpretation of the data were performed collectively by B.R., G.Z., L.C., M.N. and M.H. M.H. was responsible for drafting the manuscript which was critically revised for important intellectual content by B.R., L.C. and M.N. All authors reviewed and approved the final manuscript.

## Consent

The authors have nothing to report.

## Conflicts of Interest

The authors declare no conflicts of interest.

## Data Availability

The data that support the findings of this study are available on request from the corresponding author. The data are not publicly available due to privacy or ethical restrictions.
